# The development of postural strategies in children: a factorial design study

**DOI:** 10.1186/1743-0003-2-29

**Published:** 2005-09-30

**Authors:** Maurizio Schmid, Silvia Conforto, Luisa Lopez, Paolo Renzi, Tommaso D'Alessio

**Affiliations:** 1 Dipartimento di Elettronica Applicata, Università degli Studi "Roma TRE", Italy; 2 Unità di Neurologia Infantile, Università degli Studi di Roma "Tor Vergata", Italy; 3 Dipartimento di Psicologia, Università degli Studi di Roma "La Sapienza", Italy

**Keywords:** Postural Control, Development, Children

## Abstract

**Background:**

The present study investigates balance control mechanisms, their variations with the absence of visual input, and their development in children from 7 to 11 years old, in order to provide insights on the development of balance control in the pediatric population.

**Methods:**

Posturographic data were recorded during 60 s trials administered on a sample population of 148 primary school children while stepping and then quietly standing on a force plate in two different vision conditions: eyes closed and eyes open. The extraction of posturographic parameters on the quiet standing phase of the experiment was preceded by the implementation of an algorithm to identify the settling time after stepping on the force plate. The effect of different conditions on posturographic parameters was tested with a two-way ANOVA (Age × Vision), and the corresponding eyes-closed/eyes-open (Romberg) Ratios underwent a one-way ANOVA.

**Results:**

Several posturographic measures were found to be sensitive to testing condition (eyes closed vs. eyes open) and some of them to age and anthropometric parameters. The latter relationship did not explain all the data variability with age. An evident modification of postural strategy was observed between 7 and 11 years old children.

**Conclusion:**

Simple measures extracted from posturographic signals resulted sensitive to vision and age: data acquired from force plate made it possible to confirm the hypothesis of the development of postural strategies in children as a more mature selection and re-weighting of proprioceptive inputs to postural control in absence of visual input.

## Background

Postural control has been studied throughout a century and a half [1], and the development of balance characteristics associated with the emergence and refinement of motor control has been investigated for three decades [2]. Central Nervous System (CNS) responses and developmental changes occurring in the first years of life have been deeply studied by Assaiante [3], and Woollacott and Shumway-Cook [4]. The quantitative analysis of human movement and posture has been generally exploited on children population to study biomechanical effects on gross motor skills driven by the presence of diverse pathologies, such as Cerebral Palsy [5-8], Spinal Cord Injury [9], and Muscular Dystrophies [10,11]. Starting from the work of Williams et al [12], in more recent years researchers extended the application of quantitative posturography to fine cognitive or learning disabilities [13], autism [14,15], Developmental Coordination Disorder (DCD) [16], Attention Deficit Hyperactivity Disorder (ADHD) [17], and dyslexia [18].

Quantitative posturography can thus be applied to obtain functional markers on fine competencies and their development. For instance, a perturbation in posture with challenges such as a compliant surface [19], or a concurrent cognitive task [20], can help to enlighten possible adjustment strategies or deficiencies, or to monitor balance control variations with age [21]. However, findings obtained from other researchers show some contradictions with the above: as an example, the study of simple orthostatic posture with eyes open has been proven unsuccessful in differentiating controls from autistic patients [15], and children with DCD from controls [16]. Thus, this application field, though promising, needs to be more deeply investigated.

The quantitative analysis of postural control is generally based on data acquired by a force plate that allows one to determine the instantaneous position of the Ground Reaction Force application point, which is referred to as Centre of Pressure (CoP). Several parameters in the time and/or frequency domain [22] are then extracted from these data, or from surrogate functions derived from them [23]. Even if this technique does not allow direct detection of body oscillations, which can be estimated through the use of ad hoc motion analysis systems, the relative simplicity of the set up has encouraged researchers to consider the CoP oscillations as an indirect measure of postural sway [24].

When dealing with posturographic measures, the detection of the stabilization time after stepping on the force plate is crucial: the majority of the parameters used to define the postural ability are summary measures, and their application is based on the assumption of stationarity, in that the statistical properties of the underlying data do not significantly change over time. In presence of a transitory response to an event, such as standing up from a chair or stepping on the force plate, this assumption cannot be considered as valid. Thus the transitory response should be excluded from the analysis. By analysing the first and second order moment of the CoP trajectory, Carroll and Freedman [25] estimated this non-stationary interval to be about 20 seconds long. This assumption can be however challenged by considering that the transitory phase due to a similarly demanding perturbation, such as the Sit to Stand task, has been estimated in about 3 seconds [26]. Carpenter et al. [27] showed that the first order moment of the CoP Power Spectral Density could give insights on the duration of the transitory response.

A significant age dependence of the postural measures has been demonstrated [28,29]: from a longitudinal study, Kirschenbaum et al. [30] showed that the control strategy to maintain balance does not follow a simple linear relationship with age, but a step-like transition at the age of 6 to 8 years occurs. This hypothesis can be linked to a clear rise in normalized stability limits to adult levels at age 7, as calculated by Riach and Starkes [31] by asking children to lean as far as they could in the four directions (forward, backward, left, and right) while standing. These results suggest that, at that age, the exploratory behaviour is reached, and thus the child has to work with a new strategy, which takes into account both open loop and closed loop components of balance control. By analysing postural responses to unpredicted translations of the base of support, Sundermier et al. [32] hypothesized that the development of postural control follows the maturation of fine competencies in muscle coordination.

A variety of posturographic parameters have been shown to depend on biomechanical and anthropometric factors, such as height or weight [33], and when extracting the CoP mean amplitude on a sample population ranging from 7 to 80 years, Peterka showed no changes with age if normalization with height was performed [34].

Thus, the question remains as to whether there is any reliable marker extracted from posturographic data that can give insights on the development of balance control, and whether age significantly affects posturographic data or changes as simply the result of anthropometric factors. Aim of the present study is to investigate mechanisms involved in the development of postural stability by attempting to answer these questions.

## Methods

### Participants

148 children were selected from classes of three different grades in one primary school, after obtaining proper informed consent from parents and teachers to participate in the study. None of the children had educational needs or certified disabilities. After the collection of height and weight, they were screened with a three-sided testing procedure: Quantitative Posturography, Physical Examination for Neurological Subtle Signs (PANESS), and Teachers' Rating. For the present study, PANESS Assessment [35] and Teachers' Rating were used for inclusion criteria for the sample population, and by excluding subjects outside 10^th^-90^th ^percentile, the resulting sample size for data analysis on Quantitative Posturography was reduced to 107 children, divided into three age groups (n = 41 for Seven Years' Group, Y7, n = 38 for Nine Years' Group, Y9, and n = 28 for Eleven Years' Group, Y11). Table 1 summarizes data on participants, and Table 2 provides information on PANESS and Teachers' Rating.

**Table 1:** Population anthropometric data

Age Group	Y7	Y9	Y11
N	41	38	28
Age (yrs)	7.0 ± 0.3 (Range 6.5–7.5)	9.0 ± 0.3 (Range 8.0–9.8)	11.0 ± 0.3 (Range 10.5–12.0)
Height (m)	1.22 ± 0.06	1.34 ± 0.07	1.46 ± 0.06
Weight (kg)	25.3 ± 4.7	32.5 ± 7.1	43.1 ± 8.7
BMI (kg/m^2^)	17.0 ± 2.1	18.0 ± 2.8	20.0 ± 3.1

**Table 2:** Teachers' Rating and PANESS Assessment

Teachers' Rating		
Cluster	Definition	Score

Read and Write	reading: speed and correctness writing: tract quality and correctness oral language production (vocabulary richness and fluency and structure)	Scoring 0–3 0 is best score
Arithmetics	Arithmetics text: reading and placing numbers Arithmetcs logic: operations Sequences: understands and repeats sequences days, months, alphabets and multiplication tables	Scoring 0–3 0 is best score
Attention and Movement	Motor activity in the gym/garden: follow instructions without confusing left-right, in/out Motor activity in class: from being able to sit still, to fine movements to gross movements he cannot avoid Attention: attention span	Scoring 0–3 0 is best score
Behavior:	creativity: having many interests Social behavior: being integrated in class group and having friends Team working: following group rules Autonomy: not needing continuous instructions	Scoring 0–3 0 is best score

PANESS*		

Cluster	Definition	Score

Errors	errors on tip-toe walking errors on heel walking errors on nose-finger (right) errors on nose-finger (left)	scoring 0–3, depending on total number of errors (oscillations or falls during walking, misses or wrong fingers during other tests)
Precision	Index-little tapping on thumb (right) Index-little tapping on thumb (left) Tandem walking	sequence of movements is correct from index to little with no repetitions or misses independently of rhythm Scoring 0–3.
Rhythm	Index-little tapping on thumb (right) Index-little tapping on thumb (left) Tandem walking	the self chosen rhythm is kept during task independently of misses of repetitions. Scoring 0–3.

*Adapted from Denckla [35].

Total scores for PANESS and Teachers' Rating were obtained by summing each cluster value. Subjects were excluded if at least one total score was outside [10–90] percentile.

### Procedure

A posturographic test was performed, which consisted of 2 tests of upright stance (lasting 60 seconds each) corresponding to two different conditions: standing with eyes open (EO), and standing with eyes closed (EC). Between tests an interval of 2 minutes was allowed.

Participants were asked to select a comfortable side-by-side feet position, with their arms relaxed, then make a step forward and position themselves in the middle of the force plate, as indicated by stickers, maintaining a quiet stance. Data acquisition started immediately prior to the subject stepping on the force plate. Illumination and noise were kept under control: diffuse artificial illumination of approximately 40 lux, no remarkable fixed sound sources, experiment performed during lesson time.

Relevant force and torque components were low-pass filtered (corner frequency 20 Hz, 8^th ^order elliptical filter, stopband attenuation 80 dB at 30 Hz, attenuation slope 135 dB/octave) and fed to an AD converter (100 samples/s, DAQCard™-AI-16E-4, by National Instruments Corporation), and then processed to obtain the Centre of Pressure trajectories in both antero/posterior and medio/lateral directions, CoP = {CoP_AP_(t), CoP_ML_(t)}. The maximum of the vertical component of the ground reaction force marked the subject's stepping on the force plate.

### Feature Extraction

A set of 10 summary measures were extracted from CoP data. All of them are defined and summarized in Table 3, and denoted as Posturographic Parameters (PP).

**Table 3:** Posturographic Parameters Definition

*Posturographic Parameter*	*Acronym*	*Definition*
Mean Velocity	MV	
Mean Amplitude	MA	
Sway Area	SA	
Mean Frequency	MF	
Mean Power Frequency{AP, ML}	MPF_{AP, ML}_	
Centroidal Frequency {AP, ML}	CF_{AP, ML}_	
Frequency at 95% {AP, ML}	F95_{AP, ML}_	


*T *represents the total time for processing (30 s), and CoP_{AP, ML} _are considered as purged of their mean value		

**Figure 1:**
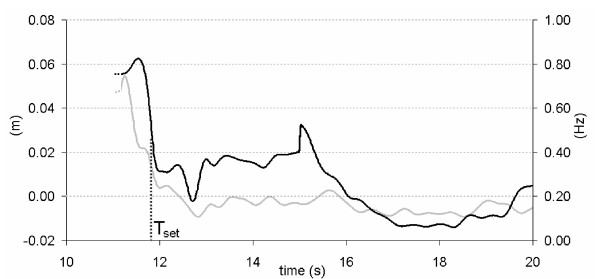
**Acquired data**. A sample of time histories for the Centre of Pressure trajectory in Antero-Posterior direction (CoP_AP_, light gray), and instantaneous mean frequency extracted from CoP_AP_. The settling time T_set _is also shown (black dotted line). All the Posturographic Parameters were calculated over the time period [T_set_, T_set _+30].

**Figure 2:**
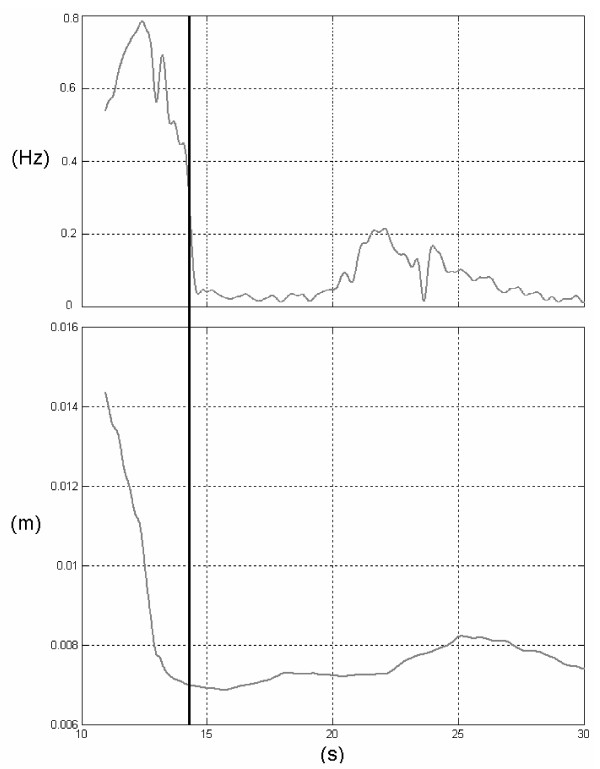
**Instantaneous Mean Frequency**. A sample of time history for the Instantaneous Mean Frequency for the Centre of Pressure Antero-Posterior (upper panel), and the Mean Amplitude value, as calculated by using 30 s starting from the corresponding time instant (lower panel). The settling time T_set _used for the actual parameter estimation is also shown (black vertical line).

A sample of processed data is represented in Figure 1. Together with the CoP_AP _trajectory over time, the time history of the corresponding instantaneous mean frequency has been depicted: Following the rationale exposed in [27], in the present work the instantaneous mean frequency (IMF) of the CoP_AP _trajectory was considered as a marker for the time needed to stabilize, its value was estimated, for every time instant t, using a complex covariance approach [36]. The settling time T_set _was then defined as the time instant when the steepest decrease of IMF occurs. This choice can be justified from experimental evidence, i.e. the behaviour of parameters object of the analysis. Using the Mean Amplitude as an example, Figure 2 shows how, after T_set_, the actual value of the parameter does not remarkably vary over time. The same applies for all the parameters object of the analysis.

All PPs were calculated by retaining the first 30 seconds after T_set_. Four of them can be directly extracted from the CoP trajectory, while the remaining six are used to characterize the shape of the Power Spectral Density: in particular, the Mean Power Frequency and the Centroidal Frequency are respectively representative of the barycentre and the dispersion of the Power Distribution in the frequency domain, i.e. the Power Spectral Density. F95% is finally representative of the overall breadth of the Spectrum.

PPs underwent statistical analysis, and, for each of them, the corresponding Romberg Ratio (RR), defined as the EC condition measure divided by the EO measure, was also computed and fed to statistics, as described in the following.

### Statistical Analysis

All PPs were analyzed through a two-way ANOVA, with vision (EO vs. EC) and age as factors. Each condition was then separately analyzed for parameters exhibiting age effect, in the following way: Bartlett's test verified homogeneity of variances, and for parameters exhibiting different variances, Welch's ANOVA was run instead of traditional ANOVA; a Post Hoc Test for trend was also applied to different age groups.

For the whole population sample, possible relationships between PPs (dependent variables) and selected subject-specific parameters (predictors) were sought to test if differences were dependent on anthropometric factors, such as body mass (m), height (h), and body mass index (BMI = m/h^2^). The linear correlation between parameters and predictors was measured through the Pearson product-moment coefficient of correlation (*r*), and deemed reliable if a two-tailed test of significance applied to this coefficient, had *p *≤ 0.05. The percentage of each PP variance that can be explained by each reliable predictor was then calculated, and denoted as σ_exp_^2^.

Then, to test changes for significant interaction between age and vision, the Romberg Ratios (RR) for each parameter underwent a one-way ANOVA, with age as factor.

## Results

**Figure 3:**
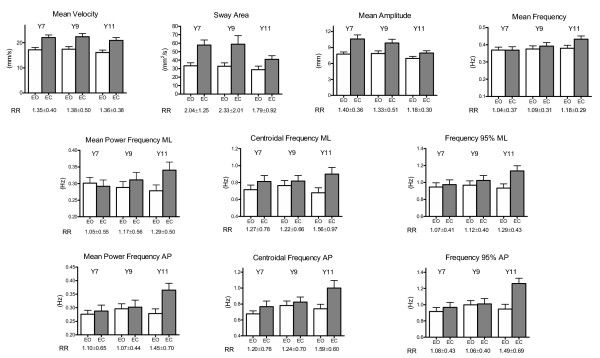
**Posturographic parameters**. Mean values and standard errors in each age group, divided by vision condition. Underneath each column pair, the corresponding Romberg Ratio mean values and standard deviation is shown.

Figure 3 summarizes sample population mean values and standard deviations for all PPs. Mean Values in EO conditions for Mean Velocity, Mean Amplitude and Sway Area were all fairly higher than those obtained on a healthy population of young adults [37]. The same did not apply to all the frequency features: Mean Power Frequency in antero-posterior (AP) direction was higher in children than in adults whereas the corresponding Centroidal Frequency was almost equal: thus, in children the CoP travelled faster, farther, and with substantially different spectral features than in adults.

As far as the differential analysis is concerned, most of the PPs were affected by vision, partly as a function of age: the effect of vision was statistically significant in MV, SA, MA, and in all the spectral parameters. This effect was more evident in amplitude parameters, thus confirming that, regardless of age, CoP displacement and velocity increased without visual input.

As reported in Table 4, age affected MA, i.e. the lower the age, the greater the CoP displacement. Moreover, two frequency parameters in AP direction, F95_AP_, and CF_AP_, were significantly affected by vision: the spectrum of CoP in AP direction was fairly broadened, even if MPF_AP _did not significantly increase. Moreover, F95_AP _was also dependent on the interaction, i.e. its variations with respect to vision were significantly different depending on age.

**Table 4:** Two-Way ANOVA p-values for posturographic parameters

PP	Age	Vision	Interaction
MV	- (0.44)	** (p < 0.001)	- (0.99)
SA	- (0.15)	** (p < 0.001)	- (0.50)
MA	* (0.014)	** (p < 0.001)	- (0.31)
MF	- (0.18)	- (0.15)	- (0.40)
MPF_ML_	- (0.82)	- (0.13)	- (0.23)
CF_ML_	- (0.89)	* (0.022)	- (0.46)
F95_ML_	- (0.42)	* (0.036)	- (0.28)
MPF_AP_	- (0.18)	* (0.046)	- (0.14)
CF_AP_	* (0.034)	* (0.013)	- (0.24)
F95_AP_	* (0.030)	* (0.009)	* (0.032)

-: Not Significant

*: p < 0.05

**: p < 0.005

Table 5 shows one-way ANOVA results for the effect of age on MA, CF_AP_, and F95_AP _in both vision conditions: Mean Amplitude did not significantly vary in EO, whereas a significant (p < 0.005) and non-random (Test for Trend p < 0.05) effect of age was revealed in EC; CoP mean deviation from its mean position actually decreased with age in no-vision condition (EC), and from Bartlett's Test it can also be speculated that the decrease in variance could be a sign of more homogeneous behaviour. The broadening of the spectrum enlightened by the previous results was principally due to the significant increase of F95_AP _with age in EC condition (Test of Trend p < 0.005), with a significant change in F95_AP _variability.

**Table 5:** Effect of age on Posturographic Parameters

PP	Age	Bartlett's Test	Test for Trend
MA (EO)	- (0.22)	* (0.046)	- (0.21)
MA (EC)	** (0.0037)	** (0.0003)	* (0.01)
CF_AP _(EO)	- (0.27)	* (0.044)	- (0.38)
CF_AP _(EC)	- (0.10)	- (0.417)	* (0.035)
F95_AP _(EO)	- (0.51)	- (0.929)	- (0.70)
F95_AP _(EC)	** (0.005)	- (0.704)	** (0.002)

- : Not Significant

* : p < 0.05

** : p < 0.005

One-way ANOVA with post hoc tests for PPs resulting in a significant effect of age, separated for vision condition: Welch ANOVA test was applied for unequal variances resulting from Bartlett's Test (i.e. on first three rows).

The correlation with anthropometric and biomechanical factors yielded the following results: only frequency parameters in EC condition, namely CF_AP _and F95_AP_, were found to be slightly dependent on mass and height, but none of them could be satisfactorily predicted by these factors (see Table 6), as the percentage of explained variance did not exceed 10% in any of them. MPF_AP _was slightly dependent on height, though the percentage of explained variance was only 4%. Thus, the confounding effect driven by the chosen anthropometric factors can be disregarded in this study.

**Table 6:** Anthropometric effect on posturographic parameters

PP	Mass		Height		BMI	
	
	p	σ_exp_^2^	p	σ_exp_^2^	p	σ_exp_^2^
MPF_AP _(EC)	- (0.061)	-	* (0.040)	4.0%	- (0.40)	-
CF_AP _(EC)	* (0.013)	5.8%	* (0.009)	6.3%	- (0.18)	-
F95_AP _(EC)	* (0.0055)	7.1%	* (0.0058)	7.0%	- (0.0945)	-

- : Not Significant

* : p < 0.05

** : p < 0.005

Regression Analysis on PP resulting in a dependence with at least one anthropometric factor. p-value, and percentage of the explained variance with the corresponding anthropometric predictor, if significant.

As a final point, the Romberg Ratios (EC/EO) revealed mean values greater than 1 for all the parameters (see Figure 3): in particular, a significant effect of age on MPF_AP _and F95_AP _was revealed, which could be the result of a significant broadening of the Power Spectral Density in EC condition in Y11. Welch's test revealed significant differences on RR variances for MPF_AP _and F95_AP _(see Table 7).

**Table 7:** Romberg Ratios: effect of age

RR	Age	Welch's Test	Test for Trend
SA	- (0.35)	- (0.30)	- (0.49)
MA	- (0.14)	- (0.053)	- (0.051)
MPF_AP_	* (0.025)	* (0.045)	* (0.020)
CF_AP_	- (0.13)	-(0.24)	- ()
F95_AP_	** (0.0012)	* (0.015)	** (0.0014)

- : Not Significant

* : p < 0.05

** : p < 0.005

One-way ANOVA p-values for Romberg Ratios, with age as factor: significance, Welch's Test for variances, and post hoc test of trend.

## Discussion

A large number of posturographic measures were sensitive to the testing condition (i.e. eyes open vs. eyes closed). If the trajectory of the CoP can be considered as an indirect measure of postural sway, and thus a marker for the control of stance, the presented results confirm the well-known thesis that visual input contribution plays a relevant role in postural stabilization. From the results on MV, SA, and MA, it is indeed possible to state that, with eyes closed, the CoP displacement and velocity increased relative to eyes open. It is known that also young adults can improve postural performance by using visual targets [38], and that closing eyes affects postural measures [22]. Ratios between EC and EO in the present study, however, were rather different from those obtained by Prieto [22] on young adults: restricting the analysis to time domain measures, thus including MF which is a surrogate parameter for time domain measures, similar ratios resulted for MV, SA, and MF. On the other hand, MA ratios tended to young adults' figures only at 11 years, while remaining higher for the other ages. For the frequency domain measures, all RR on both CF and F95 revealed higher values than young adults [22], while no comparison was possible for MPF, which is by definition different from the Median Frequency computed by Prieto. Moreover, Prieto removed very low frequency (f < 0.15 Hz) shares to spectral measures, and thus a comparison could be affected by this choice.

**Figure 4:**
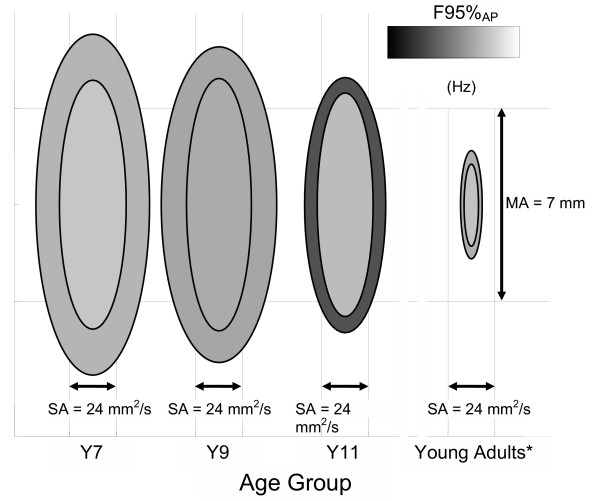
**Postural development schema**. A schematical representation of three parameters extracted from each population: the minor axis is proportional to the Sway Area, whereas the major axis is proportional to Mean Amplitude. Code luminance is proportional to F95_AP _(0.75 Hz corresponds to white, and 1.5 Hz to black). For each age group, inner ellipses turned out for Eyes Open condition, and outer ellipses for Eyes Closed. * Young adults' values are taken from Prieto et al. [22]

A graphical schema of changes in postural sway is represented in Figure 4. A non monotonous trend with age was present: the control of balance, though not to be considered complete at the last stage (Y11), was rather different from the early stages (Y7 and Y9), and confirmed the hypothesis of a nonlinear development of postural control, consistent with [30,31]. To be more specific, if the overall postural performance could be summarized through the MA measure, a clear transition occurred between 9 and 11 years. At 7 and 9 years, the possible presence of a change of strategy in EC condition did not compensate for the absence of vision, thus resulting in an overall increase of MA. At 11 years, a change on the efficacy of strategy occurred, as confirmed by the significant variations on the spectral features of the CoP trajectory, both in antero-posterior and in medio-lateral directions, which determined a significant decrease of MA RR in Y11 with respect to Y9 and Y7. The invariance of both MV and its corresponding Romberg Ratio may conceal two diverse behaviours: at 7 and 9 years, the line integral increased with occluded vision mostly due to the increase of the oscillation amplitude, while at 11 it rises because of an increase in frequency of self-sustained oscillations. Basically, when the child is younger, up to 9 years, her/his postural control with eyes closed relies on major adjustments, characterized by more ample oscillations, and the child probably needs to move to different spots and remain on those until the next adjustment. After that age, data of the present work would suggest that the child can apply minor adjustments that happen over a smaller trajectory, but with higher frequency components, as shown by the substantial increase of F95%_AP_, and there is no need for big excursions, although overall the path remains constant. The substantial increase of data variability in Romberg Ratios for F95%_AP _in Y9 with respect to Y7 and Y11 confirms the hypothesis of a change in strategy around that age. This evidence is in accordance with the hypothesis of a more mature selection and re-weighting of proprioceptive inputs to postural control: a major role of this kind of afferents could result in an increase of the high frequency contributions to postural sway [39], and thus in a broadening of the spectrum. The presented results are in accordance with the presence of a non linearity in balance control processes, as evidenced by Hay and Redon [40], who justify this step-like behaviour through the refinement of on-line control, once the feedforward mode has been efficiently developed, and by Baumberger et al. [41], who showed that the age of 10 is a critical point in the development of the visual control of stability.

## Conclusion

The obtained results are in favour of a non monotonic development of postural strategies in children, slightly dependent on anthropometric factors: the role of vision clearly varies within the studied age range, and probably the maturation of balance control is not yet complete, even at the age of 11. Finally, another question is to be unveiled: is the maturation of balance control paralleled by a corresponding change in cognitive processes? The application of dual tasks, such as a concurrent cognitive one, in the execution of quiet stance trials could help in providing information on this issue.
